# Advances in epigenetic alterations of chronic lymphocytic leukemia: from pathogenesis to treatment

**DOI:** 10.1007/s10238-023-01268-x

**Published:** 2024-03-16

**Authors:** Xin Zhang, Hua Wang, Ya Zhang, Xin Wang

**Affiliations:** 1 Department of Hematology, Shandong Provincial Hospital, Shandong University, Jinan, 250021 Shandong China; 2 Department of Hematology, Shandong Provincial Hospital Affiliated to Shandong First Medical University, Jinan, 250021 Shandong China; 3 Taishan Scholars Program of Shandong Province, Jinan, 250021 Shandong China; 4 Branch of National Clinical Research Center for Hematologic Diseases, Jinan, 250021 Shandong China; 5 National Clinical Research Center for Hematologic Diseases, the First Affiliated Hospital of Soochow University, Suzhou, 251006 China

**Keywords:** Chronic lymphocytic leukemia, Epigenetics, Mechanism, Targeted therapy

## Abstract

Chronic lymphocytic leukemia (CLL) is a heterogeneous disease with alterations in genetic expression and epigenetic modifications. In recent years, the new insight into epigenetics in the pathogenesis of CLL has been developed considerably, including DNA methylation, histone modification, RNA methylation, non-coding RNAs as well as chromatin remodeling. Epigenetic modification regulates various processes such as stem cell biology, cell growth, and tumorigenesis without altering gene sequence. Growing evidence indicates that the disturbance of gene expression profiles which were regulated by epigenetic modifications exerts vital roles in the development and progress in CLL, which provides novel perspectives to explore the etiology of CLL. In addition, the integration with epigenetic therapeutic targets and the in-depth understanding of epigenetic therapy contribute to develop new therapeutic strategies for CLL. Herein, the present review discusses the advances of epigenetic alterations in the pathogenesis, diagnosis, and prognostic assessment of CLL patients and also highlights existing and emerging agents targeting epigenetic regulators.

## Introduction

Chronic lymphocytic leukemia (CLL) is the main type of adult leukemia across western countries, which occurs in the elderly for the most part [1–3]. The CLL clinical course ranges from months to several years, which makes it a highly heterogeneous disease [4]. In more than 80% of CLL patients, genomic aberrations were detected. These chromosomal aberrations include 13q, 11q and 17p deletions, and trisomy 12 [5]. Among them, the 13q14 deletion is the most frequent abnormality [5]. Although there have been many studies on the pathogenesis of CLL and great achievements have been made in various aspects, relapse/refractory cases and drug resistance are still existing problems.

Epigenetics refers to the heritable changes in gene function with no alterations in DNA sequences, consisting of DNA methylation, histone modification, nucleosome remodeling, and so on [6]. Epigenetic modifications convey the information that plays a key role in regulating DNA-based processes [6]. During the past few decades, epigenetic modifications were found playing significant roles in the occurrence and development of leukemia by some studies and were considered a promising target for treating different types of leukemia and other hematological malignancies [7], and at the same time, it has achieved good clinical effects [8]. For example, the anti-tumor drugs azacitidine and decitabine based on inhibiting DNA methylation have significant effects in treating myelodysplastic syndrome (MDS) and acute myeloid leukemia (AML) [9].

Epigenetic researches have also made great progress in CLL, and these promote our comprehension of the pathogenesis of CLL and provide further prospects for diagnosis and treatment strategies. In the present review, we summarize recent advances of CLL in epigenetics such as DNA methylation, histone modification, RNA methylation, non-coding RNAs, and chromatin remodeling and also highlight existing and emerging drugs targeting epigenetic regulators.

## DNA methylation in CLL

**Fig. 1:**
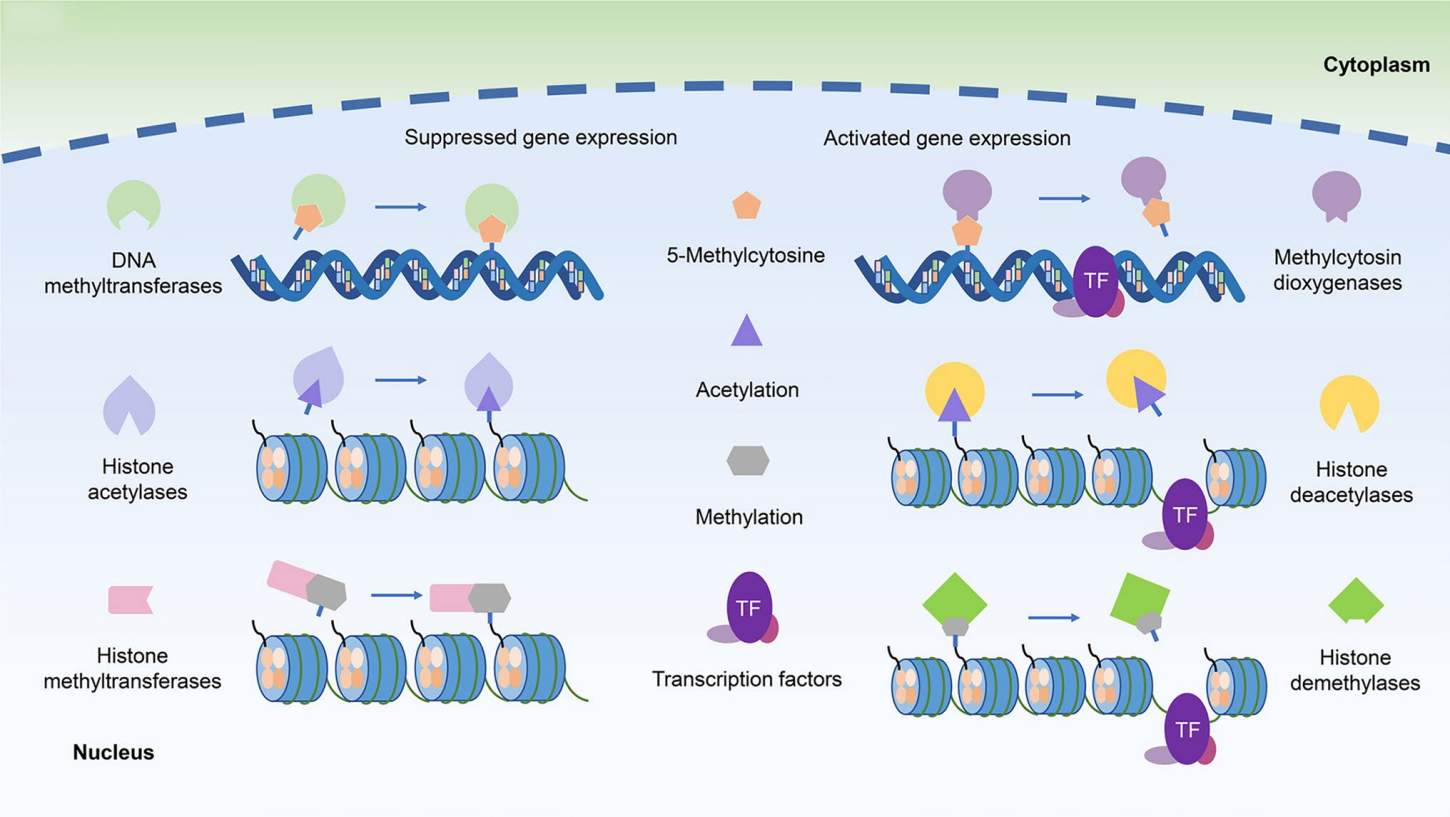
Mechanisms of DNA methylation and histone modification regulating gene expression in chronic lymphocytic leukemia (CLL)

DNA methylation is a form of chemical modification of DNA in which methyl groups are added to the C5 position of the cytosine with s-adenosylmethionine (SAM) as a methyl donor to form 5-methylcytosine under the action of DNA methyltransferases (DNMTs; Fig. 1). DNMT family of enzymes catalyze the transfer of a methyl group from SAM to DNA; three members of which have been reported possess methyltransferase activity in mammals: DNMT1, DNMT3a, and DNMT3b. Cytosine methylation is the most widely studied DNA methylation in humans.

As an essential epigenetic modification, aberrant DNA methylation has been found to be associated with an increasing number of diseases by participating in lots of cellular processes [10]. For example, increased p16^INK4a^ silencing due to DNA methylation affects cell senescence, aging, and cell cycle progression, leading to unlimited cell proliferation [11] in solid tumors such as hepatocellular carcinoma, lung cancer [12], and cervical cancer [13]. Studies suggest that silencing of cell adhesion regulators CDH1 (E-calmodulin) and CDH13 (H-calmodulin) induced by DNA methylation may promote tumor invasion and metastasis [14, 15]. In gastric and colon cancer, suppression of mismatch repair factor MLH1 expression by DNA methylation is related to genomic instability [16, 17]. DNA methylation changes in tumor usually have two aspects: global hypomethylation influencing retroviral elements and the stability of genome, and focal hypermethylation of tumor suppressor gene promoters [18].

## Global analysis of DNA methylation in CLL

Compared with healthy control, the DNA of peripheral blood mononuclear cells from patients with CLL is globally hypomethylated. Furthermore, increased mutations and genomic instability are related to less DNA methylation in genome [19]. The emergence of methyl-CpG-binding domain protein-enriched genome-wide sequencing (MBD-Seq) makes it possible to analyze methylated CpG-rich regions in the whole genome. For example, a study displayed the whole methylome of high CpG-rich regions on the basis of MBD-Seq. Compared to normal controls, 5800 hypermethylated and 12,570 hypomethylated CLL-specific differentially methylated genes were identified. Interestingly, non-coding RNA contains 40% hypermethylated genes and 60% hypomethylated genes. In addition, CpG island methylation can influence CLL based on the level of CD38 expression according to a large-scale analysis of DNA methylation [20]. Moreover, for IGHV subgroups grouping by IGHV mutational status, the proportion of common repetitions such as short interspersed elements and long interspersed elements is considerable in CLL-specific differentially methylated regions [21]. Conversely, global DNA methylation/demethylation levels assessment can improve CLL outcome prediction in patients particularly with del (13q) combined 5-methylcytosine (5-mCyt) and conventional cytogenetic approach [22].

In a population-based case–control study, the genome-wide DNA methylation of 48 CLL cases and 28 healthy controls was analyzed using the Infinium HumanMethylation450 BeadChip. In total, 34,797 differentially methylated positions (DMPs) associated with the CLL genome were identified, most of which were hypomethylated and located in gene body regions. Among them, the methylation of ZAP70, FMOD, and ADAMTS17 was significantly different between CLL cases and controls [23]. Their roles in the pathogenesis of CLL deserve further exploration.

As one of the most common mutated genes in CLL, mutations of splicing factor 3b subunit 1 (SF3B1) are associated with adverse prognosis [24]. The study to explore the connection between methylome changes and SF3B1 mutation showed that the methylation levels in 67 genomic regions in CLL patients with SF3B1 mutation were localized declined, mainly near telomeric regions [25].

## Hypermethylation and hypomethylation of single-gene promoters in CLL

In CLL, tumor suppressor genes (TSGs) expression is usually silenced by DNA hypermethylation. Wnt signaling is essential for the development of normal B-cell and has been proven to control normal apoptotic process, while abnormal activation of this pathway has been noticed in CLL [26]. Secreted frizzled-related protein 4 (SFRP4) is a member of secreted frizzled-related proteins (SFRPs) family and plays a negative regulatory role in the Wnt signaling pathway, which was observed to be often methylated in samples of CLL. Additionally, silencing of SFRP through CpG island methylation in CLL may activate the Wnt signaling pathway abnormally [27, 28]. In primary CLL samples, abnormal DNA methylation and silencing happened in SFRP4 and other SFRP family members. Through a detailed study of five SFRP family members, SFRP1 was found to be hypermethylated and down-regulated in all samples collecting from CLL patients, indicating that this epigenetic event is a key step in the development of leukemia [29]. What’s more, miR-34b/c, a type of non-coding RNA, was demonstrated as tumor suppressors, the promoter of which is abnormally hypermethylated [30]. TWIST2 is a transcription factor, whose expression is associated with the promoter methylation degree. After the treatment of decitabine, the expression of TWIST2 was increased in a CLL cell line whose promoter is methylated. Studies on 53 CLL patients showed that 72% samples from patients with mutated IGHV demonstrated TWIST2 methylation, while only 16% samples from patients with unmutated IGHV were methylated [31].

In addition to hypermethylation of tumor suppressor gene promoters, hypomethylation of oncogene promoters is also of significance in the pathogenesis of CLL. Lipoprotein lipase (LPL) plays a crucial role in pathways associated with fatty acid degradation and signaling in CLL, which might affect the behavior of CLL cells [32]. The overexpression of LPL mRNA is showed to be related to unmutated CLL status and poor clinical outcomes [33]. The reason for the abnormal expression of LPL in unmutated CLL is the demethylation of the LPL gene [34]. In mammalian cells, DNA methyltransferase 3A (DNMT3A) and DNA methyltransferase 3B (DNMT3B), which were members of DNMT3 family, are responsible for establishing DNA methylation dynamically [35]. In CLL, DNMT3A down-regulation was common. A B-cell-restricted Dnmt3a knockout mouse model demonstrated that loss of DNMT3A expression was able to drive the development of CLL and was related to aggressive disease, Notch and Myc signaling activation, and Notch inhibition sensitivity enhancement [36].

## DNA methylation profiles in different CLL subgroups

Even in CLL patients with analogous somatic hypermutation status, the DNA methylation profile may be different. Integrated analysis of DNA methylation identified that CLL stereotyped subset #8 (IGHV4-39/IGKV1(D)-39) demonstrated a unique DNA methylome compared with the other U-CLL cases, including subset #6 (IGHV1-69/IGKV3-20) and the hypomethylated and overexpressed TP63 gene becomes a pro-survival factor [37]. Through the whole-epigenome analysis of DNA methylation of CLL patients for the duration of treatment, it was found that enrichment for diverse CLL-specific epigenetic traits responded to chemotherapy that predict patient clinical outcomes, and especially involve epigenetic silencing of HOXA4 in decreasing the therapeutic sensitivity of CLL cells [38]. By comparing the DNA methylation groups of 139 CLL patients with mutated or unmutated IGHV and a couple of mature B-cell subpopulations it can be found that the two subtypes of CLL have different DNA methylation profiles that appear to represent epigenetic imprints from different normal B-cell subpopulations. The most common difference between normal B-cells and the two subtypes of CLL, and between naive B-cells and memory B-cells, is the hypomethylation of DNA in the genome that mainly targets enhancer sites [39]. Additionally, epigenetic burden and recurrent changes are correlated with specific clinical and biological characteristics according to a study of DNA methylation patterns in paired pre-treatment/relapse specimens of 34 CLL patients receiving chemoimmunotherapy, which suggests that DNA methylation responds differently to chemoimmunotherapy in patients with CLL [40].

## Histone modification

Histones and DNA together form nucleosomes, which are the main components of chromatin. Histones are proteins with highly conserved sequences in the nucleus, including 5 kinds of H1, H3, H2A, H2B, and H4. The amino acid residues on the amino terminal peptide chain of the core histones can be covalently modified under the modification of the addition or removal of already-existing methyl, acetyl, or phosphate groups by a variety of histone appearance modifying enzymes and the types of modification include methylation, acylation, phosphorylation, and others [41] (Fig. 1). Histone methylation mainly occurs in lysine and arginine residues, and the process is catalyzed by protein arginine *N*-methyltransferases (PRMTs) and histone lysine N-methyltransferases [42]. The histone acetylation of lysine has been shown highly dynamic and regulated by the opposite effects of two enzyme families, histone acetyltransferases (HATs) and histone deacetylases (HDACs). HAT utilizes acetyl CoA as a cofactor to catalyze the transfer of acetyl groups to the lysine side chain ε-amino group. HDAC enzyme antagonizes HAT and reverses lysine acetylation, which stabilizes the local chromatin structure [43].

Histone modifications alter the transcriptional state of chromatin and transform it into euchromatin with higher transcriptional activity or heterochromatin with lower transcriptional activity. Thus, histone modification affects the development of related diseases by regulating gene transcription and translation. In the CD19 + B-cells of CLL patients, the overexpression of SIRT1 and EZH2, global histone H3/H4 hypoacetylation, and H3 K9 hypermethylation were detected, which indicated that the abnormal histone modification played key roles in the pathogenesis of CLL [44].

## Histone methylation

Histone methyltransferases (HMTs) are essential in regulating gene transcription, which can transfer methyl groups to histone proteins from SAM [45]. In solid and hematological malignancies, HMT is destroyed by mechanisms such as chromosomal translocations, genome loss, and/or point mutations [46]. Many HMT aberrations were found in human malignancies. Among them, the repeated deletion and/or inactivating mutations of SETD2, a cancer suppressor gene, originally found in renal clear cell carcinoma [47]. SETD2 is able to catalyze the trimethylation of lysine 36 on histone 3 (H3K36me3), which is one of the main chromatin marks related to active transcription. Evidence supporting the tumor suppressor effect of SETD2 is that its deletion impairs DNA repair and enhances genome precariousness [48, 49]. SETD2 abnormity is a frequent, early loss-of-function event associated with aggressive disease in CLL pathology. Through high-resolution single nucleotide polymorphism (SNP) arrays, repeated loss of the SETD2 locus was identified in 3% of CLL patients. The loss of SETD2 was related to TP53 deletion, genomic complexity, and chromothripsis. In five chemotherapeutic or chemoimmunotherapeutic clinical trials, compared with cases whose three genes are wild type, patients with SETD2 aberrations and wild-type TP53 and ATM had poorer progression-free survival (FPS) and overall survival (OS) [50].

Enhancer of zeste homolog 2 (EZH2) is a human homolog of Drosophila zeste gene enhancer 2, which mainly inhibits the activity of target genes or silences target genes directly through histone modification, thus regulating cell senescence, differentiation, tumorigenesis, and development [51]. Increased expression level of EZH2 relates to an unfavorable prognosis in CLL [52]. A study demonstrated the elevated expression of EZH2, c-Myc, E2F1, and pRb proteins and decreased miR-26a expression in the proliferation centers (PCs) of CLL/small lymphocytic lymphoma (SLL) [53]. A large prospective CLL trial cohort showed that elevated KDM1A and associated gene expression signatures related to aggressive disease and dismal prognosis. Integrated analyses of differential global transcriptomes and H3K4me3 marks in E*µ*-TCL1A vs. iKdm1aKD; E*µ*-TCL1A mice implied KDM1A as an oncogenic transcriptional repressor in CLL by altering histone methylation patterns with obvious effects on defined cell death and motility pathways [54].

## Histone acetylation

Histone acetylation is a reversible dynamic equilibrium process, which is mediated by two enzymes, histone acetyltransferase (HAT) and histone deacetylase (HDAC). Generally, histone acetylation is related to gene expression activation, while histone deacetylation tends to down-regulate gene expression. In CLL leukemia cells, the E-cadherin gene is hypoacetylated. The transcription of this silent gene can be activated by treating with histone deacetylase inhibitor (HDACi) MS-275. Compared with the aberrant exon11 skipped transcripts, the more rightly spliced E-cadherin transcripts expressed by activated genes can inhibit the Wnt signaling pathway [55]. Histone acetylation and other epigenetic modifications jointly promote the occurrence and development of CLL.

## RNA methylation

**Fig. 2:**
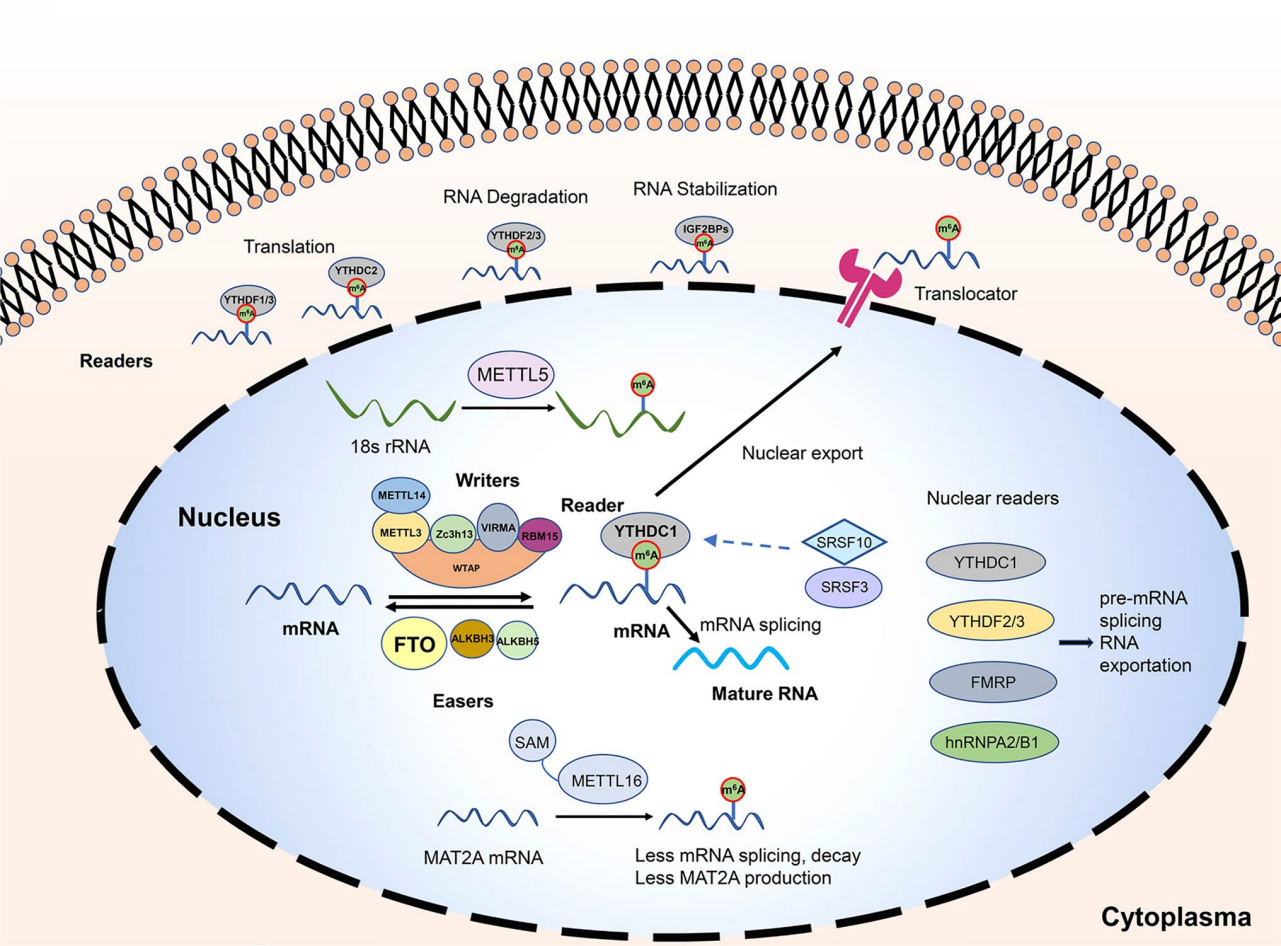
The mechanism of m^6^A RNA methylation in eukaryotic cells

RNA methylation is of great significance in regulating gene expression. N^6^-methyladenosine (m^6^A) RNA methylation modification is the main type of mRNA modification [56]. M^6^A methylation involves methyltransferases (writers) and m^6^A-binding proteins (readers), which can be reversed by demethylases (erasers; Fig. 2). Methyltransferases mainly include methyltransferase-like 3 (METTL3), methyltransferase-like 14 (METTL14), Wilms tumor 1-associated protein (WTAP), and methyltransferase-like 16 (METTL16) which mediates the process of RNA methylation. The METL3/METTL14/WTAP complex is directly engaged in the methylation regulation of nuclear splicing-related organelles [57]. METTL16 regulates the activity of RNA spliceosome by regulating the methylation level of small nuclear RNA (snRNA) in nucleus [58]. Demethylases include fat mass and obesity-associated protein (FTO) and alkylation repair homolog 5 (ALKBH5), which mediate the procedures of RNA demethylation. Demethylases can directly locate to nuclear speckles, which are rich in splicing factors and closely related to the splicing process of mRNA, and participate in the regulation of methylation. The most common m^6^A-binding proteins are the YTH domain-containing family 1–3 (YTHDF1-3) and YTH domain-containing protein 1–2 (YTHDC1-2), which can recognize the RNA methylation modification and take part in downstream RNA translation, degradation, splicing, and other processes [59]. Evidence showed that m^6^A modification is associated with tumorigenesis, tumor multiplication, aggression, and metastases [60, 61].

There was evidence that FTO, a m^6^A mRNA demethylase, contributes to the development and progress of AML [62, 63]. Studies have demonstrated that FTO expression was upregulated in CLL patients and was related to a poor prognosis. Moreover, FTO accelerates the survival of CLL cells through DNA damage pathway. A novel inhibitor selectively targeting FTO, FB23-2, has an effective therapeutic potential in eliminating cell survival and inducing cell cycle arrest through m^6^A methylation [64].

## Non-coding RNA

### MicroRNA

MicroRNAs (miRNAs) can regulate post-transcriptional silencing of target genes which are short RNA molecules with a size of 19–25 nucleotides. Additionally, a single miRNA usually affects the expression of lots of genes by involving in a functional interacting pathway [65]. The expression and functional characterization of these miRNAs are listed in Table 1.

**Table 1:** The expression and functional characterization of non-coding RNAs in chronic lymphocytic leukemia

	Name	Expression	Function	References
MicroRNAs	MiR-15/16	Down	Induce the cell death via inhibiting the expression of Bcl2 and the regulation of TP53	[62]
	MiR-34a/b/c	Down	Inhibit the cell proliferation and associated with the expression of TP53 and ZAP-70	[66]
	MiR-155	Up	Enhances B-cell-receptor (BCR) signaling and is associated with poor prognosis	[69, 70]
	MiR-29b	Down	Modulates CD40 signaling by targeting TARF4	[72]
	MiR-125a-5p/miR-34a-5p	Up/down	Valuable markers to predict Richter syndrome (RS) development	[73]
	MiR-150	Down in worse prognosis	Modulate heterogeneity via regulating expression of GAB1 and FOXP1	[74]
LncRNAs	CRNDE	Down	Inhibit cell proliferation via miR-28/NDRG2 axis and regulated by DNA methylation	[80]
	Lnc-LEF1-AS1	Up	Interacts with LEF1 and promotes Cell proliferation	[81]
	AC092652.2–202	Not reported	Related to ultraconserved region 70 and has prognostic significance	[82]
	Lnc-TOMM7-1	Down-regulated in early stage	Antisense to the interleukin-6 (IL6) gene and promotes cell death	[83]
	Lnc-KIAA1755-4	Up	Involvement in ribosome formation and translational processes	
	Lnc-IRF2-3	Up in IGHV-unmutated	Associated with primary immunodeficiency and has prognostic significance	
	LincRNA-p21	Up in TP53^wt^	Induces the death of cell after DNA damage in a p53-dependent manner	[85]
	NEAT1	Up in TP53^wt^	Involvement in DNA damage and transcriptive process activated by p53	[85]
CircRNAs	Circ-RPL15	Up	Increases cell proliferation and diagnostic biomarker	[89]
	Circ-CBFB	Up	Increases cell proliferation and prognostic and diagnostic marker	[90]
	Circ_0132266	Down	Promotes cell viability through miR-337-3p/PML axis	[91]
	CircZNF91	Up	Promotes the Malignant Phenotype by Targeting the miR-1283/WEE1 Axis	[92]
	Circ_0002078	Up	Promotes cell proliferation by regulating the expression of TCF7L1	[93]
	Mc-COX2 (Mt-circRNAs)	Up	Inhibits apoptosis and promotes cell proliferation	[94]

**Fig. 3:**
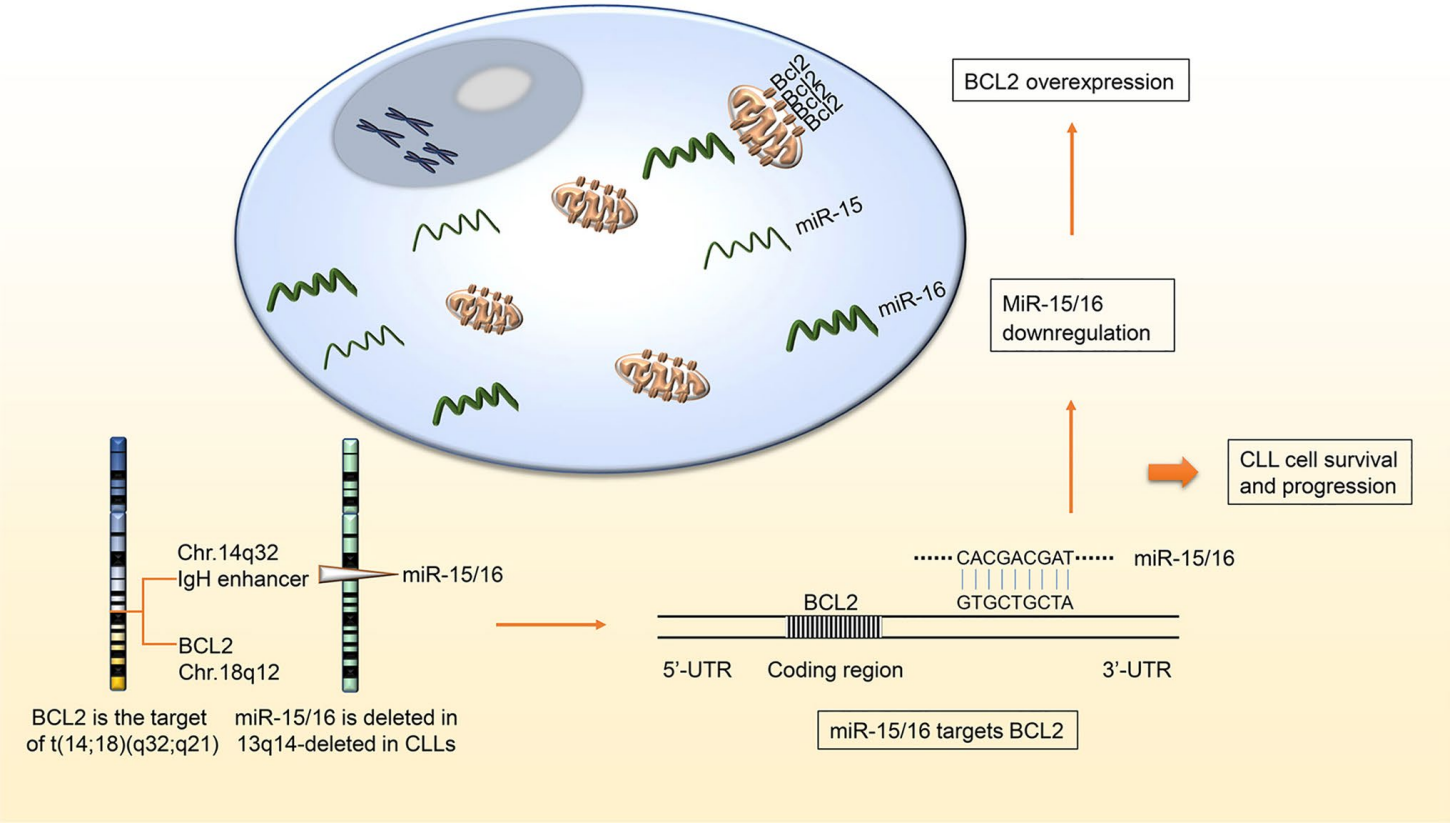
The mechanisms of miR-15 and miR-16 regulate cell apoptosis by targeting BCL2 in CLL

The loss of miR-15/16 gene on chromosome 13q14 is the most common alteration in CLL. In a small region of chromosome 13q14, miR15 and miR16 locate at the translocation breakpoint that is deleted in more than 65% of CLL. What’s more, in this region, allelic loss is associated with down-regulation of miR-15/16 expression which demonstrate that allelic loss leads to the inactivation of these genes in CLL [66]. Remarkably, the first 9 nucleotides in the 5′-ends of miR-15/16 and bases 3287–3279 in the 3′-end of the BCL2 cDNA are complementary sequences, which was found overexpressed in nearly all CLL patients [67]. Much more, the interaction between these miRNAs and BCL2 is direct [68]. The mechanism by which miR-15/16 regulates the expression of BCL2 and promotes apoptosis is shown in Fig. 3.

MiR-34b and miR-34c at chromosome 11q negatively regulate protein synthesis. Furthermore, the clinical process of the cases with deletion of chromosome 11q usually showed aggressive. The miR-34b/c promoter region was found to have high frequency of abnormal hypermethylation, which was highly in relation to the level of miR-34b/c and the deletion of 11q, confirming miR-34b/c has become a new potential anti-oncogene on chromosome 11q23 [30, 69].

MiR-155 as a critical regulator involved in the B-cells post-transcriptional gene expression is evolutionarily conserved encoded in a region which is called the B-cell integration cluster (BIC, miR155HG) [70]. The high-level miR-155 expression can enhance the sensitivity to BCR ligation. Besides, the cross-talk of the tissue environment can induce the overexpression of miR-155. These findings potentially contribute to connect miR-155 with adverse clinical outcomes in CLL patients [71, 72].

There are also some other miRNAs reported in the past decade, such as miR-29 (proved to have an anti-tumor effect by targeting TRAF4 which can be affected by BCR inhibitors and selectively delivered to CLL cells [73, 74]), miR-125a-5p/miR-34a-5p (as valuable markers to predict Richter syndrome (RS) development in CLL patients [75]), and miR-150 (much abundant expressed in CLL and attached to disease outcome via GAB1 and FOXP1 [76]). A recent research based on forty newly diagnosed CLL patients shows that miRNAs might be the prognostic biomarkers in the process of CLL and endpoint predictors in this disease. Some researchers also found some CLL-delivered exosomes internalized by stromal cells can deliver miRNAs, which can induce an inflammatory phenotype in target cells similar to that of cancer-associated fibroblasts [77].

### Long non-coding RNA

Long non-coding RNA (lncRNA) is a kind of RNA with a length of > 200 nucleotides, which is least or no potential possibility to encode proteins [78]. Deletion of chromosome 13q region is considered to be the most common chromosomal abnormal observed in CLL which contains two lncRNAs (DLEU1 and DLEU2). The former is known as the host gene for miR-15 and miR-16 [79]. Thus, deletion of 13q14 leads to inactivation of both collaborating tumor suppressor genes, DLEU7 and miR-15/16. MiR-15/16 and DLEU7 inactivation, respectively, lead to increased BCL2 expression and induction of TNF signaling by TRAFs [80]. There are some lncRNA candidates examined by several studies listed in Table 1, which contains an overview of the functional characterization of these lncRNAs across CLL [81–85].

### The association between the p53 and lincRNA-p21

Studies have shown that the deletion of P53, which is related to adverse prognosis in CLL patients, can regulate the expression of some lncRNA. A study showed that in primary CLL cells with wild-type TP53, lincRNA-p21 was up-regulated after radiation, resulting in decreased cell viability, while cells with TP53 mutations or deletions lack this mechanism [86, 87]. The transcription of lincRNA-p21 can be activated by p53 through binding the promoter of lincRNA-p21, and the lincRNA-p21 can also regulate the activity of p53 [88, 89].

### Circular RNA

As important members of the gene regulatory environment, circular RNAs (circRNAs) are another kind of non-coding RNA. CircRNA-miR combination has been demonstrated to regulate transcriptome distribution and cell function in physiological and pathological processes; however, the role of circRNAs in cells remains to be explored [90]. To date, five main circRNAs circ-RPL15 [91], circ-CBFB [92], circ_0132266 [93], circZNF91 [94], and circ_0002078 [95], as well as mitochondrial genome-derived (mt)-circ-RNAs mc-COX2 [96] have been focused in CLL (Table 1).

For CLL patients without IGHV mutation, CircRPL15 is considered as a latent biomarker for the diagnosis in plasma [91]. The high expression level of circRPL15 is supposed to increase RAF1 protein levels by sponging miR-146b-3p. In proliferative RAS pathway, RAF1 is an effector which can promote cell growth by phosphorylating and activating mitogen-activated protein kinase (MAPK) signal. [97]. Through the research of its mechanism, it was found that circ-CBFB-activated Wnt/ *β*-Catenin pathway inhibits the production of Wnt receptor frizzled 3 (FZD3). Circ_00002078 was found highly expressed in CLL and can inhibit cell apoptosis, promote cell proliferation and cell cycle arrest through miR-185-3p/TCF7L1 axis [95]. Besides, recently a study showed that the expression of mc-cox2 in mitochondria is up-regulated in plasma and exosomes of CLL patients and may be involved in disease progression [96].

### Chromatin remodeling

Chromatin remodelers consist of four families: SWI/SNF (SWItch/Sucrose Non-Fermentable), ISWI (Imitation of SWItch), CHD (Chromodomain Helicase DNA binding), and INO (INOsitol), which are subdivided by the core ATPase subunit and involved in many pathological processes in CLL. A couple of mutations involving chromatin remodeling have been found to affect the ARID1A and CHD2 genes in hematological malignancies, including CLL [98].

ARID1A is a tumor suppressor, which can contribute to the SWI/SNF chromatin remodeling complex formation and has been indicated to interact directly with p53 [99]. In addition, CHD2 is a member of the SNF2-adenosine triphosphate (ATP-dependent) chromatin remodeling factor CHD family. CHD2 is a complex multi-domain protein consisting of n-terminal tandem chromogenic domains (chromatin tissue modification domains) followed by DEXDc (death-like spirase superfamily) domains and HELIC (C-terminal of the spirase superfamily) domains, both spanning SNF2 (N-terminal of the SNF2 family) domains. CHD2 also contains a putative DNA binding domain (DBD) and a C-terminal domain (DUF4208) of unknown function [100]. Studies have confirmed that the C-terminal part of CHD2 is a functional DBD, which is selective for double-stranded DNA and is necessary to stimulate the ATPase and chromatin remodeling activities of the protein [101].

## Epigenetic-targeted therapy

### Demethylation agents

DNA methylation is catalyzed by DNMTs, the inhibitors of which can inhibit that process [102]. Azacitidine, a DNMT inhibitor, is not therapeutically effective in CLL, and a phase II clinical trial of azacitidine in fludarabine-refractory CLL was terminated prematurely due to lack of response and slow recruitment [103]. CLL cells can cause abnormal immune regulatory mechanisms that favor T-cell dysfunction and immunosuppression, with an inability to present antigens to the T-cell arm of the immune system. One study combining two epigenetic modifiers 5-aza-2'-deoxycytidine and histone deacetylase inhibitors (HDACis) LAQ824 was effective in restoring immunogenicity in CLL cell lines as well as in primary cells obtained from CLL patients [104].

### HDACis

As a kind of promising noval epigenetic anticancer agent histone deacetylase inhibitor (HDACi) can induce a variety of biological process in cancer cells including gene expression regulation, G1/S or G2/M cell cycle arrest, differentiation, and apoptosis [105, 106]. AR-42 (Arno Therapeutics) targeting Class I and IIB HDAC enzymes is an orally bioavailable small molecule, which has anti-tumor activity in solid tumor models in vitro and in vivo [107] and many B-cell malignancies [108]. In CLL cells, AR-42 can not only increase the sensitivity of CLL cells to TNF-related apoptosis inducing ligand (TRAIL) by reducing the expression of cellular FLICE (FADD-like IL-1β-converting enzyme)-like inhibitory protein (c-FLIP), but also engender dose and time-dependent acetylation of histone, thereby inducing apoptosis from dependence on caspase [109].

According to an analysis of apoptosis regulatory genes in CLL, Kendine 92, and SAHA, HDACis have been confirmed that both of them can induce dose-, time- and caspase-dependent apoptosis via the mitochondrial pathway [110]. Ms-275, an HDACi, has been reported to mediate its cytotoxic effects by producing reactive oxygen species (ROS) in proliferating hematopoietic cell lines [111]. Another one is the basic DEPSIPTED (FR901228) clinical trial of DEPSIPTED (FR901228) CLL that is an early observation that CLL has selective in vitro activity in cultured CLL cells (0.038-micron DEPsipeptide). DEPsipeptide induces acetylation of histone H3 and H4 and inhibits deacetylation at concentrations comparable to LC50 [112]. The acetylation of histone occurs to H4 K5, H4 K12, and H3 K9 most commonly, and then H4 K8, but no H4 K16 or H3 K14, which are lysine specific.

However, not all HDACis exhibit efficient anti-tumor activity. MGCD0103 as an orally available class I HDACi, has limited activity in monotherapy in high-risk CLL patients [113]. Much more, preclinical studies of the HDACi DEPsipeptide (FK228) in CLL demonstrated that it can effectively induce apoptosis and at concentrations where HDACi occurs. Although FK288 effectively inhibits HDAC in CLL patients, its use in the current administration plan is limited because of progressive physical symptoms [114].

### DAPK inhibitor

The death-associated protein kinase 3 (DAPK3), which mediates histone phosphorylation and responds to the BCR signaling pathway activation, is recruited to RNA polymerase II in an anti-IgM-dependent manner. DAPK inhibitors do not inhibit transcription on its own, but affect mRNA processing, and have a wider antitumor activity than ibrutinib through inhibiting anti-IgM and CD40L-dependent activation [115]. Targeting DAPK3 is a promising alternative for the treatment of CLL with BTK inhibitors.

## Conclusions

Altogether, we summarized the recent advances in epigenetics mechanisms and targeted therapies of CLL. Better and earlier identification of the exact function of epigenetic alterations including DNA methylation, histone modification, RNA methylation, non-coding RNAs, and chromatin remodeling can provide new insights into the etiology of CLL. Although there are few mature epigenetic regulators, targeting epigenetics is still one of the directions for the future treatment of CLL.

## Data Availability

Not applicable.
